# The Free-Running Circasemilunar Period Is Determined by Counting Circadian Clock Cycles in the Marine Midge *Clunio Marinus*

**DOI:** 10.1177/07487304241249516

**Published:** 2024-05-19

**Authors:** Jule Neumann, Dharanish Rajendra, Tobias S. Kaiser

**Affiliations:** * Max Planck Institute for Evolutionary Biology, Plön, Germany; † Julius-Maximilians-Universität Würzburg, Würzburg, Germany

**Keywords:** circalunar clock, T-cycle, counter hypothesis, oscillator hypothesis, beat hypothesis

## Abstract

Semilunar rhythms are found in numerous marine organisms, but the molecular mechanism and functional principles of endogenous circasemilunar clocks remain elusive. Here, we explore the connection between the free-running circasemilunar clock and the circadian clock in the marine midge *Clunio marinus* with three different chronobiological assays. First, we found that the free-running circasemilunar period of the adult emergence rhythm in *C. marinus* changes linearly with diel T-cycle length, supporting a day-counting mechanism. Second, under LD 6:6, periods of circasemilunar and circadian emergence were comparable to those under LD 12:12, indicating that the circasemilunar counter in *C. marinus* relies on endogenous circadian oscillations rather than external T-cycles. Finally, when desynchronizing the circadian clock with constant light, the free-running circasemilunar emergence rhythm disappeared as well, suggesting that it requires a synchronized circadian clock. These results oppose the long-held view that *C. marinus’* free-running circasemilunar clock operates independently of the circadian clock. In a broader evolutionary context, our results strengthen the idea that the circasemilunar clocks of dipterous insects are based on different functional principles compared to the circasemilunar or circalunar clocks of marine annelids and algae. These divergent clock principles may indicate multiple evolutionary origins of circasemilunar and circalunar clocks.

Life on Earth evolved under recurring environmental cycles, namely the tides (T = 12.4 h), night and day (T = 24 h), the lunar cycle (T = 29.5 days), and the seasons (T = 1 year). In anticipation of the predictable conditions that accompany these geophysical cycles, organisms have evolved corresponding endogenous time-keeping mechanisms—so-called biological clocks. In marine habitats, especially in the intertidal zone, organisms are constantly exposed to dramatic changes in the environment governed by the tides (e.g., salt concentration, UV radiation, temperature). Adaptation to those is reflected in the prevalence of circatidal clocks (Rock et al., 2022). In addition, the tidal amplitude is modulated over the semilunar cycle (T = 14.77 days), reaching its maximum during the spring tides around full moon and new moon. To synchronize reproduction or behavioral rhythms such as locomotion and feeding to the most favorable tidal amplitude, organisms use circasemilunar and circalunar clocks (Naylor, 2010). The endogenous nature of circa(semi)lunar clocks has been validated across the eukaryote branch of the tree of life, in algae, cnidarians, annelids, mollusks, crustaceans, insects, and fish (Kaiser and Neumann, 2021). However, their molecular mechanisms remain unknown (Andreatta and Tessmar-Raible, 2020). Three hypotheses for the functional principle of circasemilunar clocks have been put forward (Bünning and Müller, 1961): a molecular oscillator with an intrinsic 15-day period (oscillator hypothesis), a day-counting mechanism (counter hypothesis), or superposition of a circadian and a circatidal rhythm, which results in a 15-day beat phenomenon (beat hypothesis). These hypotheses can be tested with specific chronobiological experiments, without the need for molecular readouts or tools. However, out of more than 20 species with a known circasemilunar clock, the functional principle has only been examined for four of them (Kaiser and Neumann, 2021). We argue that in order to understand the molecular mechanism, it is important to know the operating principle of the clock because it guides the search for molecular readouts and candidate genes.

One experiment to distinguish between the beat, counter, and oscillator hypotheses was proposed by Bünning and Müller in the early 1960s. By experimentally manipulating the diel T-cycle length, the free-running circasemilunar period is expected to change differently depending on the operating principle of the clock (Figure 1a). The free-running period of a 15-day circasemilunar oscillator that operates independently of the circadian system should be unaffected by changes in T-cycle length. In contrast, a semilunar day-counting mechanism must go through a fixed number of “steps” and, therefore, depends on either the endogenous circadian period or the length of the exogenous environmental T-cycle. This is based on the assumption that the circadian period can be modified by the superimposed diel T-cycle because the circadian clock can maintain a fixed phase angle with the T-cycle within its ranges of entrainment (Aschoff, 1978). Hence, the semilunar period is expected to change linearly with T-cycle length under the counter hypothesis. Finally, the pattern of the semilunar beat wave depends on the underlying circadian and circatidal periods. Because of the non-linear interaction of the two, slight changes in the T-cycle are expected to drastically change the semilunar period (Figure 1a).

**Figure 1.**
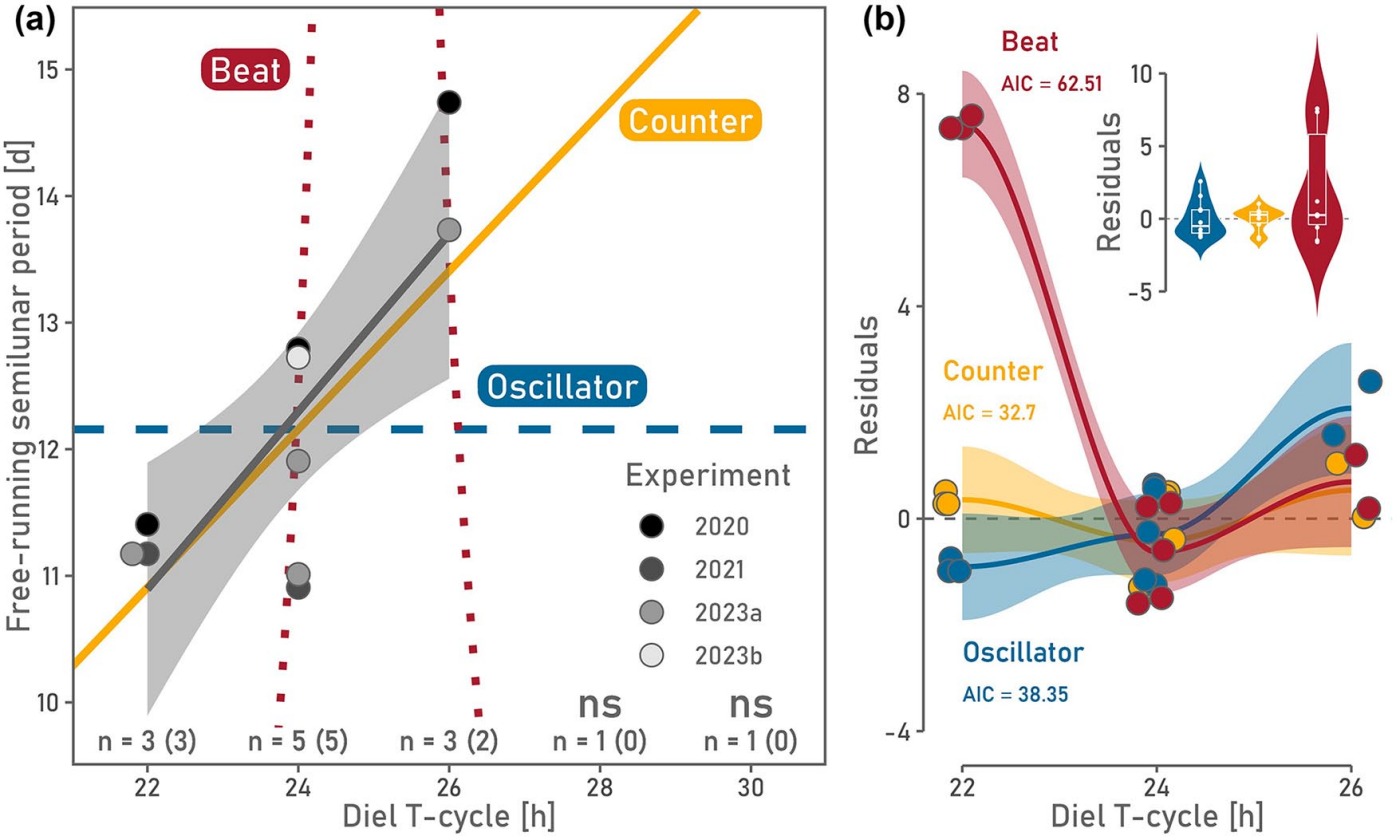
Observed circasemilunar periods of *C. marinus’* emergence rhythm under different T-cycle lengths are compared with periods as predicted by three hypotheses (counter = yellow, oscillator = blue, and beat = red). (a) Period of the free-running circasemilunar emergence rhythm increased linearly with T-cycle length between 22 and 26 hours. Emergence is arrhythmic under T-cycles of 28 and 30 hours. *n* represents the number of replicates conducted. The number of significant values used for downstream analyses is depicted in brackets. The linear model fitted to the observed periods is depicted as the gray solid line (ci = 0.95). Overlapping periods observed under the T-cycle of 22 hours were jittered along the x-axis for better visualization. Replicates are labeled according to the year they were conducted. 2023b refers to the control of the constant light experiment. (b) The residuals (difference between observed and expected periods) are plotted for all three hypotheses. The AICc value was the lowest for the counter hypothesis, indicating that it is the best-fitting model. All residuals were jittered along the x-axis for visualization of overlapping data.

We investigated the functional principle of the circasemilunar clock in the marine midge *C. marinus* (Diptera, Chironomidae). *C. marinus* inhabits the intertidal zones along the rocky shores of Europe (Neumann, 1966). A circasemilunar clock controls the semilunar adult emergence rhythm, which restricts the emergence and reproduction of the short-lived (few hours) adults to the spring tides, when eggs can be deposited on the exposed larval substrates (Neumann, 1988). The circadian clock both governs the time of the day when adults emerge and sets a nocturnal sensitivity window for the perception of moonlight cues (Neumann, 1989). The beat hypothesis has been convincingly ruled out for *C. marinus* by the experiment proposed earlier conducted for T-cycles of 23.2, 24, and 24.4 hours (Neumann, 1976). However, these slight differences in T-cycle length are not informative for distinguishing between the counter and oscillator hypothesis. The oscillator hypothesis has been favored for the semilunar emergence rhythm of *Clunio*, based on the observation that the rhythm continues in constant light (LL) (Neumann, 1976) and constant darkness (DD) (Neumann, 1988), ruling out the counting of light-dark (LD) cycles (Neumann, 1989). However, these experiments do not exclude the possibility that *Clunio* counts endogenous circadian oscillations. Such a mechanism has been suggested for the marine midge *Pontomyia oceana* (Soong and Chang, 2012).

We monitored the free-running circasemilunar adult emergence rhythm of *C. marinus* under different diel T-cycle lengths ranging from 22 h to 30 h to capture the change in the circasemilunar period predicted by the counter hypothesis. We found that the circasemilunar period of *C. marinus’* emergence rhythm changes linearly with T-cycle length, supporting the counter hypothesis. In order to further distinguish a counting mechanism rooted in the circadian system from one based on external T-cycles, we exposed *C. marinus* to a T-cycle of 12 h (LD 6:6). The circadian clock is an oscillatory system that, when exposed to an environmental cycle having a submultiple of its period, can entrain to every second or third (or fourth, etc.) cycle, essentially maintaining a 24-h period. This phenomenon is known as frequency demultiplication (Wever, 1960). The period of a circasemilunar counter based on endogenous circadian oscillations should be unaffected by diel T-cycles being a submultiple of 24 h. In contrast, if the circasemilunar counter takes information directly from the environmental T-cycle, its period is expected to halve under LD 6:6 compared to LD 12:12. In our experiment, both the circasemilunar and the circadian period remained unchanged under LD 6:6 compared to LD 12:12, suggesting the circasemilunar rhythm in *C. marinus* is based on counting circadian clock cycles. Finally, in the experiment conducted under LL by Neumann (1976), the emergence rhythm was monitored for less than 30 days. Hence, this is not enough time to capture the truly free-running circasemilunar period. To test if *C. marinus* requires a synchronized circadian clock for maintaining the semilunar emergence rhythm, we repeated the experiment and exposed midges to LL for more than 80 days and monitored both the circadian and circasemilunar emergence rhythm. The results confirm that *C. marinus’* day-counter mechanism is based on counting endogenous circadian cycles.

## Methods

### Laboratory Cultures and Circasemilunar Entrainment

A laboratory strain of *C. marinus* collected from Helgoland, Germany, was reared at 18°C in plastic containers filled with natural North Sea water and deionized water (1:1) according to a standard protocol (Neumann, 1966). Animals were supplied twice a week with diatoms (*Phaeodactylum tricornutum*; strain UTEX646) and every 2 weeks with powdered nettles (*Urtica sp.*). The circasemilunar emergence rhythm was entrained by a 24-h LD cycle and mechanical vibrations given in a tidal pattern of T = 12.4 h using an unbalanced motor (50 Hz, ~ 30 dB above background noise, motor alternated between 6.2 h on and 6.2 h off; Neumann and Heimbach, 1979; Neumann, 1978). Air temperature and light intensity were measured and recorded every 10 minutes using Onset HOBO data loggers (UA-002-08) for all experiments.

### T-Cycle Experiment

Three independent experimental runs were conducted in 2020, 2021, and 2023 with the Helgoland laboratory strain of *C. marinus*. The experiment consisted of two phases: During the *entrainment phase*, animals were reared from eggs under LD 12:12 and tidal vibration. During the subsequent *experimental phase*, the semilunar entrainment was concluded (no tidal vibration), and larvae were exposed to T-cycles of differing lengths: T = 22 h (LD 11:11), T = 24 h (LD 12:12), T = 26 h (LD 13:13), T = 28 h (LD 14:14), or T = 30 h (LD 15:15). Changing the diel T-cycle length while the circasemilunar clock was free-running ensured that the introduced changes in T-cycle length did not re-entrain the circasemilunar clock. Larvae were between 2 and 8 weeks old when entering the experimental phase. The mixed age structure was necessary because the circadian and circasemilunar phenotypes can only be observed at the population level. Emergence occurs only once during the insects’ lifetime. However, multiple emergence peaks are required to calculate the circasemilunar period reliably. Hence, different age cohorts of the population are required. In total, five time series replicates were conducted for T = 24 h, three for T = 22 h and T = 26, and one for T = 28 h and T = 30 h (Supplementary Figures S1-S5). For the T = 24 h treatment in 2021, a phase shift in the emergence rhythm occurred (Supplementary Figure S1b, gray arrow), which coincided with heavy vibrations due to construction works at our institute. However, the number of emergence peaks is sufficient to calculate the circasemilunar period reliably. We included an additional time series of T = 24 h, which was the control to the LL experiment for downstream analysis (see section “Constant light (LL) Experiment,” Figure 5, labeled 2023b in Figure 1). The circasemilunar phenotype is the number of emerged adults counted once during the first half of the light phase of each T-cycle treatment. Numbers were assigned to the 24-h day on which the preceding LD transition occurred because this is when midges emerged (Neumann, 1966; Neumann and Heimbach, 1985). T-cycle lengths longer than 24 h results in information ostensibly missing on some 24-h days. Circasemilunar emergence data can be found in Supplementary File S1 (2020, 2021, 2023a, 2023b). The time series for calculation of the circasemilunar period started on day 31 of the experimental phase (explanation see section “Time Series Analysis”) and lasted until day 112 (82 days in total) for all experimental runs. For shorter time series, missing data were encoded as NA.

### LD 6:6 Experiment

Both the circadian and circasemilunar phenotypes were assessed under LD 6:6 in *C. marinus*. Similar to the T-cycle experiment, the setup consists of two phases. For assessment of the circadian phenotype, *C. marinus* was entrained with tidal vibration under LD 16:8. Subsequently, 4- to 6-week-old animals were transferred into either LD 6:6 or LD 12:12 and semilunar free-run (no tidal vibration). Emergence was recorded hourly using a custom-made fraction collector (Honegger, 1977). The middle of the night was defined as zeitgeber time (ZT) 0, and data were adjusted accordingly. The transfer into the new light regime was defined as day 0 of the time series. Time series analysis started with the first full day in LD 6:6 (day 1). The length of the circadian time series was 91 days (hourly data, 2184 bins). Circadian emergence data are provided in Supplementary File S2 (2022). For assessment of the circasemilunar phenotype, a population of mixed age was entrained under LD 12:12 and tidal vibration as previously described. Two replicates were conducted in 2021 and 2023. Semilunar time series started with day 31 of the experimental phase and lasted until day 112 (82 days in total). Circasemilunar emergence data are provided in Supplementary File S1 (2021, 2023a).

### Constant Light (LL) Experiment

*C. marinus* midges were entrained with tidal vibration and LD 12:12. Larvae were between 4 and 6 weeks of age when the circasemilunar clock was released into free-run conditions (no tidal vibration) and LD 12:12. During the experimental phase, midges were released either into LL (~1000 lux) or kept in LD 12:12 as a control, and the hourly number of emerged midges was monitored as described earlier. The transfer into LL was defined as day 0 of the time series. Circadian time series analysis started with the first full day in LL (day 1). The length of the circadian time series was 83 days (hourly data, 1992 bins). Circadian emergence data are provided in Supplementary File S2 (2023). The semilunar time series started on day 31 after being released into circasemilunar free-run and lasted until day 112 (82 days in total). Circasemilunar emergence data are provided in Supplementary File S1 (2023b).

### Time Series Analysis

The first 30 days after midges were released into circasemilunar free-run were excluded for calculation of the circasemilunar period because eclosion behavior in *C. marinus* is developmentally predetermined up to 20 days in advance (Neumann and Spindler, 1991), that is, midges emerging during the first 20 days in circasemilunar free-run were already developmentally bound to emerge at a specific time and were therefore not affected by the treatment. We removed the first two full semilunar cycles (30 days) in order not to include partial emergence peaks. All statistical analyses were conducted in R version 4.3.0 (R Core Team, 2023).

To visualize periodic patterns in the circasemilunar time series of the T-cycle experiment, auto-correlation was calculated using the acf() function with *na.action* *=* *na.pass*. Periods were visualized using Lomb-Scargle (LS) periodograms (Ruf, 1999), calculated with lsp() and *alpha* *=* *0.05, type* *=“period,” from* *=* *3, to* *=* *35* for circasemilunar time series and *alpha* *=* *0.05, type* *=“period,” from* *=* *15, to* *=* *40* for circadian time series. Circasemilunar and circadian periods for downstream analyses and model selection were calculated using the meta2d() function combining the Jonckheere-Terpstra-Kendall (JTK) and LS algorithms as part of the MetaCycle package v 1.2.0 (Wu et al., 2019). Integrated period (meta2d period) is calculated as the arithmetic mean of periods calculated by JTK and LS. The integration of multiple algorithms allows a more robust estimation of the period compared to using a single one. The ARSER algorithm available in MetaCycle cannot be used for time series containing missing data and was therefore excluded. For circasemilunar periods, parameters were set to *minper* *=* *3, maxper* *=* *20, outIntegration* *=“onlyIntegration,” cycMethod* *=* *c(“LS,” “JTK”), combinePvalue* *=“fisher.”* The boundaries were chosen because they cover all periods expected under the different hypotheses. All circasemilunar time series were analyzed together (n = 16, day 31-112 of circasemilunar free-run) and can be found in Supplementary File S1. The corresponding R script is available as Supplementary File 3. For circadian time series, parameters were set to *minper* *=* *20*, *maxper* *=* *28*, *cycMethod* *=* *c(“LS,” “JTK”)*, *combinePvalue* *=“fisher.”* For time series longer than 1000 time points, JTK could not calculate a period. Hence, for the circadian time series of the LL and LD 6:6 experiments, we only report the periods calculated by the LS periodogram. All circadian time series can be found in Supplementary File 2. The corresponding R script is available as Supplementary File 4. Circadian time series of the same length were analyzed together. Integrated periods from meta2d() were considered significant when the *p*-value of meta2d was <0.05 (corrected by the Benjamini-Hochberg procedure).

### Model Selection and Fit

To test which of the hypotheses fit the observed circasemilunar periods best, we fit three different models, corresponding to the counter, the oscillator, and the beat hypotheses. We compared the residuals, Akaike information criterion for small sample sizes (AICc), and AICc weight of each of them. All calculations are based on the observed circasemilunar periods (τ) computed with meta2d(). The residuals were retrieved with res() and calculated as:

(1) $$\tau_{observed}-\tau_{expected}$$

Under the counter hypothesis, we expect a linear relationship between T-cycle length (T) and circasemilunar period (τ) . Hence, we fit a linear model for T-cycle lengths of 22 h, 24 h, and 26 h in R with:

(2) lm(τ∼T)

Under the oscillator hypothesis, the circasemilunar period is *not* expected to change with T-cycle length. Hence, the slope of the regression line is 0, and the expected circasemilunar period is calculated as the mean of all observed periods. We therefore encoded the oscillator model in R as:

(3) lm(τ∼1)

The expectation for the beat hypothesis of an entrained, semilunar 15-day period in hours is calculated as:

(4) $$\tau_{beat}=\frac{T*k}{2*|T-k|}$$

whereby *T* is T-cycle length in hours, and *k* is the duration of the lunar day in hours (k=24.8h) . The semilunar period in days is calculated as:

(5) $$\frac{\tau_{beat}}{24}$$

However, k=24.8h holds only true for the entrained semilunar period. The free-running circasemilunar period deviates from 15 days. Hence, the formula needed adjustment. We assume that the tidal parameter *k* takes a different but fixed value. This is based on the assumption that a potential circatidal oscillator free-runs unaffected by T-cycles while the circadian period is modified by them. The best-fitting value for *k* was calculated using nls() with *start* *=* *24.8*. Expected periods under the beat hypothesis were then calculated with the estimate k=25 (estimate = 25.0292, *p* ≤ 2e-16) for obtaining the residuals. The beat model was encoded in R as:

(6) $$\begin{array}{l} beat.model<-function(T,k)\\ \left((T*k/\left(2*abs(T-k))\right)/24\right) \end{array}$$

(7) nls(τ∼beat.model(T,k))

To compare the fit of the models, the second-order AICc was calculated with AICc() of the MuMIn package version 1.47.5 (Bartoń, 2023). The weights of the AICc values were calculated with akaike.weights() of the qPCR package version 1.4-1 (Spiess, 2018).

## Results

### The Free-Running Circasemilunar Period Increases Linearly With T-Cycle Length in *C. marinus*

To distinguish between oscillator, counter, and beat hypotheses as outlined in the introduction, we monitored the semilunar emergence rhythm of *C. marinus* in circasemilunar free-run and diel T-cycles of 22, 24, 26, 28, and 30 hours (Figure 1a). Circasemilunar rhythmicity was clearly evident for T-cycles between 22 h and 26 h, and only for those T-cycles significant free-running periods were obtained with meta2d() (Supplementary Figures S1-S3). Thus, we assessed the different hypotheses based on the time points in Figure 1a.

The expectation for the circasemilunar period under the beat hypothesis does not match the observed periods. Under a T-cycle length of 22 h, residuals for the beat hypothesis deviate vastly (Figure 1b). In line with that, the AICc value and residual standard error were the highest for the beat model compared to the counter and oscillator models (Table 1). The beat hypothesis can, therefore, be ruled out, which is in line with previous experiments (Neumann, 1976). This leaves the counter and the oscillator hypotheses for *C. marinus*. When plotting the residuals for the oscillator and counter hypotheses (Figure 1b), values are close to 0 for both. However, the residual standard error was lower for the counter model (0.842) than for the oscillator (1.304), indicating that the former fits better than the latter (Table 1). A lower AICc value and higher Akaike weight also support the counter as the best-fitting model (Table 1; 32.70 vs 38.35). The linear model corresponding to the counter hypothesis fits the data significantly (estimate = 0.702, *p* = 6.12e-03, gray solid line, ci = 0.95). The slope of the linear model (0.702) is very close to the expected slope under the counter hypothesis (0.625). These results favor the counter hypothesis over the oscillator hypothesis.

**Table 1.** Selected models and parameters for the beat, counter and oscillator hypothesis.

Hypothesis	Model	Residual standard error	AICc	A. weight
Counter	lm(τ∼T)	0.842	32.70	9.44e-01
Oscillator	lm(τ∼1)	1.304	38.35	5.58e-02
Beat	nls(τ∼beat.model(T,k))	4.365	62.51	3.17e-07

The smallest residual standard error, the lowest AICc value, and the highest Akaike weight indicate that the counter hypothesis fits the observed circasemilunar periods better than the oscillator and beat hypothesis.

There is a second line of evidence supporting the counter hypothesis: If the molecular machinery underlying *C. marinus’* emergence rhythm was driven by an oscillator with an intrinsic 15-day period, it should be unaffected by changes in diel T-cycle length in its free-running state. We would, therefore, expect a detectable (and constant) circasemilunar period also under very long T-cycles of 28 or 30 hours. However, there was no significant rhythmicity under T-cycles of 28 h and 30 h, speaking against the oscillator hypothesis. Under the counter hypothesis, if the counter relied on external T-cycles, the circasemilunar period is also expected to persist regardless of T-cycle length. However, if the counter was based on information coming from the endogenous circadian system, essentially the circadian period, long T-cycles could lie outside the circadian clock’s limits of entrainment, and therefore, the circasemilunar rhythm would be expected to disappear. It is known that diel T-cycles longer than 28 hours are outside the range of entrainment for the circadian clock in *C. marinus* (Neumann and Heimbach, 1985). Arrhythmicity observed under T = 28 h and T = 30 h, therefore, supports the counting of endogenous circadian oscillations rather than the counting of external T-cycles. To further test the assumption of counting endogenous circadian periods, we performed experiments under a T-cycle of 12 hours (LD 6:6), as well as under LL.

### The Circadian Clock Frequency Demultiplies Under LD 6:6 and the Circasemilunar Period Remains Close to 15 Days

**Figure 2.**
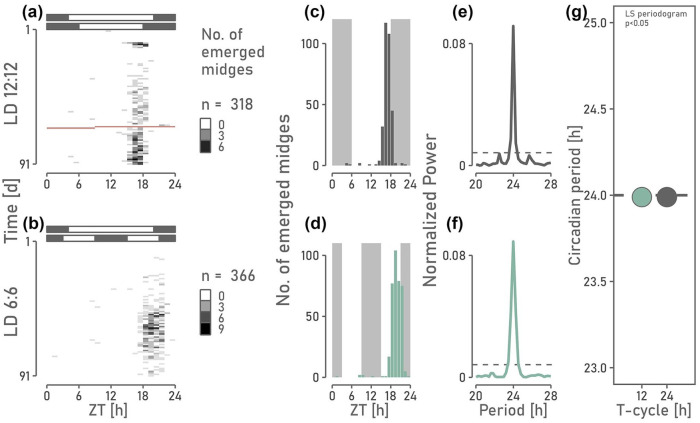
Circadian emergence phenotype of *C. marinus* under LD 12:12 and LD 6:6 after entrainment in LD 16:8. The experiment was conducted in 2022. (a, b) Hourly number of emerged midges for 91 days in (a) LD 12:12 and (b) LD 6:6. Missing values (NA) are displayed in red. (c-d) Numbers of emerged midges were summed per hour over all days. (c) Emergence occurred right before the light-dark transition in LD 12:12. (d) Emergence occurred only at one of the two light-dark transitions in LD 6:6, indicating frequency demultiplication of the circadian clock. (e-g) Lomb-Scargle periodogram analysis reveals a 24-h period of emergence under both LD 12:12 and LD 6:6.

To further investigate if *C. marinus’* circasemilunar counter is indeed counting circadian periods rather than T-cycles, we examined the circasemilunar and circadian emergence rhythm under a T-cycle of 12 h (LD 6:6). Because T-cycle length of 12 h is a submultiple of 24 h, the circadian clock may frequency demultiply, that is, may continue to run with a period close to 24 h. Indeed, the circadian emergence rhythm of *C. marinus* had the same period under LD 12:12 $\left(\tau_{circadian}=\;23.99h,\;p\;=\;1.24e-45\right)$ and LD 6:6 $\left(\tau_{circadian}=23.99h,p=6.61e-45\right)$ (Figure 2e-2g), supporting frequency demultiplication of the circadian oscillator.

**Figure 3.**
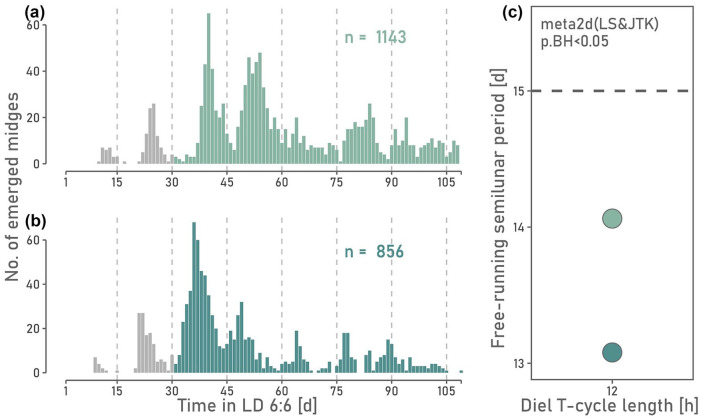
(a, b) Daily number of emerged midges for *C. marinus* under LD 6:6 and circasemilunar free-run after circasemilunar entrainment in LD 12:12. Experiments were conducted in 2021 (a) and 2023 (b). For time series analysis, only the colored bars were considered. (c) The circasemilunar period remains close to 15 days (dashed line) under LD 6:6 for both replicates (a): $\tau_{circasemilunar}=14.06d,\;p=5.73e-04$ ; (b): $\tau_{circasemilunar}=13.08d,\;p=3.16e-02.$

If the counter receives information directly from the external T-cycle, the circasemilunar period is expected to halve under LD 6:6 compared to LD 12:12. However, if the circasemilunar counter relies on counting circadian periods, the circasemilunar period is expected to stay close to 15 days under LD 6:6. This based on the assumption that the circadian period remains 24 hours through frequency demultiplication. Indeed, the circasemilunar period departed not more than 2 days from the entrained 15-day period (Figure 3), indicating that the circasemilunar counter in *C. marinus* relies on counting the circadian period, that is, endogenous information from the circadian system.

### The Circadian and Circasemilunar Clocks Desynchronize in Constant Light

**Figure 4.**
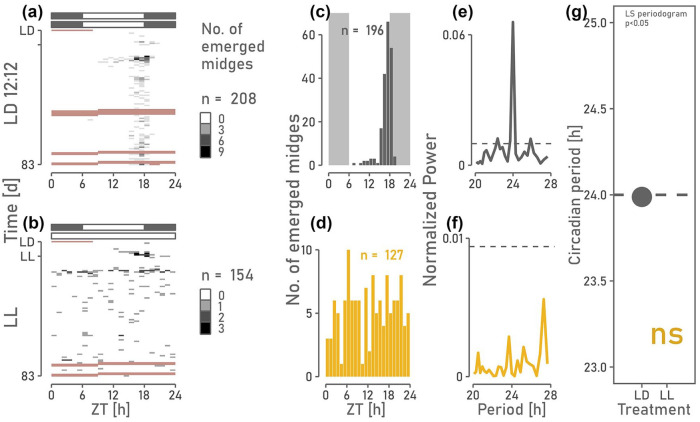
Circadian emergence of *C. marinus* in LD 12:12 and under constant light. (a) Under LD 12:12 midges emerge synchronized in phase around ZT 18, at the light-dark transition. (b) In constant light, midges emerge throughout the 24-h day. (c, d) Number of emerged midges per ZT was summed up over all 83 cycles to visualize the phase of emergence. (e-g) Lomb-Scargle periodogram analysis identifies a significant 24 h-period under LD 12:12 (e) but not under LL (f), suggesting that *C. marinus’* circadian clock desynchronizes in constant light.

**Figure 5.**
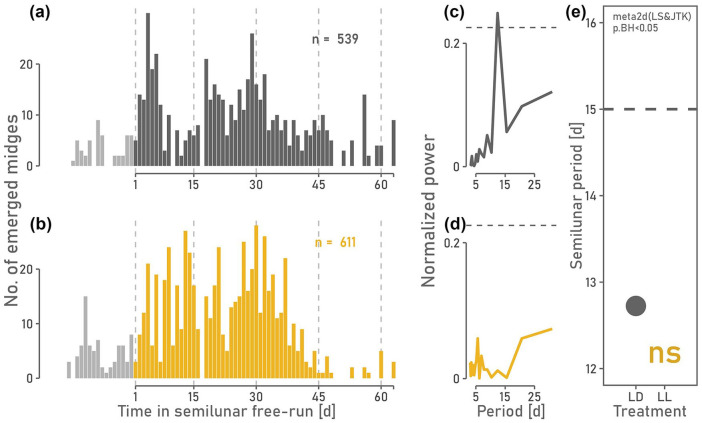
The circasemilunar emergence pattern of *C. marinus* becomes arrhythmic under constant light. (a) Number of emerged midges per day under LD 12:12. (b) Number of emerged midges under LL. (c, d) Lomb-Scargle periodogram analysis reveals that free-running circasemilunar emergence was rhythmic under LD 12:12 but not in constant light. (e) meta2d() detects a significant circasemilunar period only under LD 12:12.

The preceding experiments suggest that *C. marinus* relies on a circasemilunar counter mechanism, which relies on counting circadian clock periods. Therefore, desynchronizing the circadian clock should result in arrhythmic semilunar emergence. Constant light can lead to arrhythmicity of behavioral rhythms governed by the circadian clock of mammals (Pittendrigh and Daan, 1976) and Drosophila (Skopik and Pittendrigh, 1967), given sufficient intensity (Pittendrigh, 1966). Therefore, we exposed *C. marinus* to constant, high-intensity light and monitored the circadian and circasemilunar emergence rhythm. The constant light experiment can distinguish between the counter and oscillator hypotheses. A truly independent circasemilunar oscillator should be unaffected by a desynchronized circadian clock, unlike a counting mechanism rooted in the circadian system. Circadian emergence (Figure 4) was rhythmic under LD 12:12 $\left(\tau_{circadian}=\;23.99h,\;p\;=\;2.90e-27\right)$ but became arrhythmic under LL (*p* = 7.76e-01). Hence, the LL conditions were sufficient to desynchronize the circadian clock. If a synchronized circadian clock is essential for the circasemilunar clock, the circasemilunar emergence rhythm is expected to disappear in LL as well. Indeed, emergence was rhythmic in LD 12:12 ( $\tau_{circasemilunar}=12.72,\;p\;=\;4.67e-02$ ; Figure 5a, 5c, 5e) but arrhythmic in LL (*p* = 1; Figure 5b, 5d, 5e), indicating that a synchronized circadian clock is required for maintaining a free-running circasemilunar rhythm.

## Discussion

### *C. marinus* Counts Endogenous Circadian Oscillations for Semilunar Time-Keeping

This study characterizes the functional principle of the circasemilunar clock in the marine midge *C. marinus* by distinguishing between the beat, counter, and oscillator hypotheses. The free-running period of circasemilunar emergence increased linearly with T-cycle length between 22 and 26 h, following predictions by the counter hypothesis. Arrhythmicity under T-cycles of 28 and 30 h indicates that the counter relies on the circadian system. Consistently, under LD 6:6, the circasemilunar period was comparable to the one under LD 12:12, suggesting that the circadian clock frequency demultiplies and that the counter depends on the circadian period rather than external T-cycles. In LL, the circadian and circasemilunar periods fade, suggesting that a synchronized circadian clock is essential for circasemilunar time-keeping in the marine midge.

Until this study, the free-running circasemilunar emergence rhythm of *Clunio* was postulated to run independently of the circadian system (Neumann, 1988), favoring an independent circasemilunar oscillator as the functional principle of the circasemilunar clock. This assumption was based on two experiments in which the circasemilunar emergence rhythm continued for *C. marinus* in LL (Neumann, 1976) and *Clunio tsushimensis* in DD (Neumann, 1988). The author concluded that external LD cycles are not required for maintenance of the circasemilunar emergence rhythm and, therefore, reject the counter hypothesis. However, these experiments do not rule out that the circasemilunar counter could instead be based on endogenous circadian oscillations. If this was the case, we would have to assume that the circadian clock remains synchronized during LL and DD. For the experiment under LL, we were able to retrieve the circadian time series data from Neumann’s unpublished data. We found that in his experiment, indeed the free-running circadian period remained rhythmic during the first semilunar emergence peak ( $\tau_{circadian}=\;25.10h,\;p=0,\;day\;in\;LL\;1-7,$ hourly data, 168 bins) as well as the second semilunar emergence peak ( $\tau_{circadian}=26.83h,\;p=2.33e-06,\;day\;in\;LL\;13-19,$ hourly data, 168 bins; Supplementary Figure S6). Circadian emergence data are provided in Supplementary File S2 (1976). This LL experiment, therefore, does not rule out the counter hypothesis and does not contradict our results. Interestingly, in our own LL experiment, the circadian clock was already desynchronized after a few days, and so was the circasemilunar rhythm. We assume that this might be due to very high light intensities in our experiment. Importantly, circadian emergence in *Clunio* is a phenotype that can be observed only on the population level. Hence, we cannot distinguish if high light intensities cause the circadian clock to desynchronize on the population level, that is, circadian clocks of individual midges run out of phase, or if the molecular machinery of the circadian clock of each individual becomes arrhythmic, as seen in Drosophila (Price et al., 1995).

In agreement with our study, the beat hypothesis has previously been rejected for *C. marinus* in two experiments. First, under shorter and longer T-cycles (LD 11.6:11.6 and LD 12.2:12.2), the circasemilunar period did not vary according to expectations of the beat hypothesis (Neumann, 1976). Second, a phase shift in the LD cycle did not affect the circasemilunar period (Neumann, 1969). In the latter experiment, the circasemilunar clock of *C. marinus* (Helgoland strain) was entrained by the superposition of an LD cycle with a tidal vibration pattern. Both cycles resume a unique recurring phase relationship every 15 days. Then, the tidal vibration pattern was concluded (circasemilunar free-run), and a phase shift in the LD cycle was introduced. It was assumed that if *Clunio’s* circasemilunar clock is explained by the beat hypothesis, the now free-running circatidal oscillator would, in combination with the shift in the LD cycle, result in a phase shift of the circasemilunar emergence rhythm. However, the emergence rhythm continued without a phase shift, rejecting a beat mechanism. In fact, to this day, a tidal rhythm (and, for that matter, a circatidal clock) has never been observed in *C. marinus*, raising the question if it at all exists in the marine midge.

### Connecting the Oscillator, the Counter Mechanism and Photoperiodism in Clunio

Distinguishing between a counter and an oscillator mechanism might be an oversimplification and might not be meaningful for the biology of every organism. The circasemilunar emergence rhythm of *Clunio* is, as a whole, clearly an oscillatory system, matching all criteria for a biological clock (Aschoff, 1978), including free-run, entrainment, and temperature compensation (Neumann, 1988, 1966; Neumann and Heimbach, 1984; Neumann, 1978). However, our study suggests that the circasemilunar oscillation is not simply based on an (molecular) oscillator with a corresponding period of 15 days (oscillator hypothesis, definition by Bünning and Müller (1961)). Instead, circadian periods are counted in a step-wise manner. We must assume that the timing system can only move to the next step when a specific signal from the circadian system is received. The phase of the rhythm is set by semilunar cues. The phase response curve for the circasemilunar emergence rhythm of *C. tsushimensis* suggests an immediate resetting without transients, indicating a strong coupling between the pacemaker and the overt behavior, at least in the examined experimental conditions (Kaiser and Neumann, 2021). The absence of transient cycles also speaks against a 15-day oscillator mechanism and is in line with the idea of a counter, which can be reset immediately. Arrhythmicity of emergence in LL further supports that the circasemilunar clock depends on regular input by the circadian system.

The finding of a counter mechanism rooted in a synchronized circadian system as the functional principle for circasemilunar time-keeping in the marine midge *C. marinus* prompts a series of exciting new questions: How many “steps” or “days” are counted (semilunar 15 vs lunar 30)? How is this number determined at the molecular level? And why is the free-running circasemilunar period shorter (12-13 days) than the semilunar period in its entrained state (15 days)? Resetting by semilunar time cues can make the entrained period shorter, but how can it be lengthened to 15 days? Do semilunar cues modulate the length of the “steps”? First insights into counter mechanisms come mainly from photoperiodism research. Numerous organisms measure day or night length and express a photoperiodic response after a defined number of cycles, which seems to be temperature compensated (Saunders, 1966, 1971; Goryshin and Tyshchenko, 1970). Different models exist on how a counter could work by accumulating a signal or substance, until ultimately crossing an internal threshold leading to the phenotype (Goryshin and Tyshchenko, 1974; Gibbs, 1975; Nunes and Veerman, 1982). However, the molecular mechanism of the photoperiodic counter is not understood. The role of the circadian clock for photoperiodism, on the other hand, has been proposed a long time ago (Bünning, 1936, 1960) and, since then, has been frequently investigated, revealing that it does play a role for some but not all organisms (Saunders, 2021). Hypotheses from research on photoperiodism might provide a starting point for understanding the circasemilunar counter in *C. marinus*. It could be a candidate to find accumulating or degrading molecules (transcripts, proteins, hormones) over the semilunar cycle.

### The Functional Principle of Clunio’s Circasemilunar Clock Lays the Foundation for Unraveling Its Molecular Mechanism and for Studying Time-Keeping Across Various Time Scales

The evidence presented here establishes a link between the circadian clock and circasemilunar time-keeping in *C. marinus*. However, in order to understand the underlying molecular mechanism and substantiate a causal link between the circadian clock and the circasemilunar counter, it will be necessary to conduct functional validation studies. This requires establishing the molecular architecture of the circadian clock in *C. marinus* and subsequent genetic manipulation of core circadian clock genes. Another approach could be to chemically inhibit components of the circadian clock, as has been successfully undertaken in other non-model clock species (Zhang et al., 2013; Zantke et al., 2013). Finally, *Clunio’s* diversity of circasemilunar and circalunar emergence phenotypes makes it a unique model to study the circasemilunar counter using genomic approaches (Kaiser et al., 2016; Kaiser, 2014). In whole-genome screens, core circadian clock genes have been identified as associated with the presence and absence of circasemilunar rhythms (Fuhrmann et al., 2023). The circadian clock gene *period* was identified as a candidate involved in determining the phase of a circalunar rhythm by a combination of QTL mapping and whole-genome screens (Briševac et al., 2023). The current study provides a mechanistic framework for those genomic findings, explaining how the circadian clock could be involved in circasemilunar time-keeping of *C. marinus*.

To precisely coordinate physiological processes, organisms unite molecular mechanisms operating on different time scales, reaching from seconds to years. However, an organism’s fitness depends on the accurate adjustment of all of them. Understanding the interrelation of time-keeping systems remains challenging. Because most organisms displaying short- and long-term non-24 h rhythms are non-model organisms, bespoke molecular techniques are scarce. However, a robust hypothesis on the functional principle underlying each time-keeping system is an important first step toward understanding temporal organization in its entirety. The marine midge *C. marinus* has an experimentally verified circadian clock, as well as a circasemilunar clock and a photoperiodic diapause response (Neumann and Krüger, 1985), providing an opportunity to address the complex organization of time-keeping within the same organism.

### Divergent Clock Principles May Indicate Multiple Evolutionary Origins of Circasemilunar Clocks

A systematic investigation of the functional principles of the circa(semi)lunar clock is challenging because only a few circasemilunar clock model organisms exist, and experimental evidence is scarce (Kaiser and Neumann, 2021). Weak evidence for the beat hypothesis has been established for the alga *Dictyota dichotoma* (Bünning and Müller, 1961; Vielhaben, 1963). The semilunar rhythm of foraging behavior in the marine isopod *Scyphax ornatus* likewise shows a beat phenomenon. When the T-cycle lengths of the diel and tidal cycles were systematically manipulated, the period of the overt behavioral rhythm changed as expected under the beat hypothesis (Cheeseman et al., 2017). However, the experiment was carried out under permanent entrainment by both diel and tidal cues. Therefore, it is not clear if the animals responded to the exogenous beat of the superimposed zeitgeber cycles or to an endogenous beat of corresponding clocks. Additional free-run experiments are required to investigate this open question. The lunar maturation rhythm of the marine annelid *Platynereis dumerilii* continued when the circadian clock was chemically inhibited, suggesting that its circalunar clock does not depend on the circadian system and is, therefore, most likely based on a 30-day circalunar oscillator (Zantke et al., 2013). The lunar phase response curve of the marine annelid *Syllis prolifera* shows transient cycles, characteristic of an oscillatory system (Franke, 1986). A circasemilunar counter based on the circadian system is evident for the marine midges *C. marinus* (this study) and *Pontomyia oceana* (Soong and Chang, 2012). Thus, the circa(semi)lunar clock seems to rely on different functional principles in different organisms, which is in stark contrast to the well-conserved molecular mechanism of the circadian clock (Dunlap, 1999; Kotwica-Rolinska et al., 2021). Different functional principles of circa(semi)lunar time-keeping could be rooted in the different life histories of diverse organisms. Polychaetes are mainly marine organisms that originated during the Cambrian (Morris and Peel, 2008) and stayed in the ocean thereafter. They might have an evolutionarily ancient circa(semi)lunar clock. In contrast, marine midges (Diptera) seem to rely on a counting mechanism. Considering that the first Diptera appeared during the Middle Triassic (around 240 million years ago) in terrestrial habitats (Misof et al., 2014), they secondarily colonized the ocean. Hence, marine midges may have co-opted an already existing day-counting mechanism—the photoperiodic counter—in order to track the (semi)lunar cycle. Further investigations are required to test the idea that marine annelids and marine midges rely on different functional principles of their circa(semi)lunar clock.

## Supplemental Material

sj-docx-1-jbr-10.1177_07487304241249516 – Supplemental material for The Free-Running Circasemilunar Period Is Determined by Counting Circadian Clock Cycles in the Marine Midge Clunio Marinus Supplemental material, sj-docx-1-jbr-10.1177_07487304241249516 for The Free-Running Circasemilunar Period Is Determined by Counting Circadian Clock Cycles in the Marine Midge Clunio Marinus by Jule Neumann, Dharanish Rajendra and Tobias S. Kaiser in Journal of Biological Rhythms

sj-jpg-4-jbr-10.1177_07487304241249516 – Supplemental material for The Free-Running Circasemilunar Period Is Determined by Counting Circadian Clock Cycles in the Marine Midge Clunio Marinus Supplemental material, sj-jpg-4-jbr-10.1177_07487304241249516 for The Free-Running Circasemilunar Period Is Determined by Counting Circadian Clock Cycles in the Marine Midge Clunio Marinus by Jule Neumann, Dharanish Rajendra and Tobias S. Kaiser in Journal of Biological Rhythms

sj-jpg-5-jbr-10.1177_07487304241249516 – Supplemental material for The Free-Running Circasemilunar Period Is Determined by Counting Circadian Clock Cycles in the Marine Midge Clunio Marinus Supplemental material, sj-jpg-5-jbr-10.1177_07487304241249516 for The Free-Running Circasemilunar Period Is Determined by Counting Circadian Clock Cycles in the Marine Midge Clunio Marinus by Jule Neumann, Dharanish Rajendra and Tobias S. Kaiser in Journal of Biological Rhythms

sj-jpg-6-jbr-10.1177_07487304241249516 – Supplemental material for The Free-Running Circasemilunar Period Is Determined by Counting Circadian Clock Cycles in the Marine Midge Clunio Marinus Supplemental material, sj-jpg-6-jbr-10.1177_07487304241249516 for The Free-Running Circasemilunar Period Is Determined by Counting Circadian Clock Cycles in the Marine Midge Clunio Marinus by Jule Neumann, Dharanish Rajendra and Tobias S. Kaiser in Journal of Biological Rhythms

sj-jpg-7-jbr-10.1177_07487304241249516 – Supplemental material for The Free-Running Circasemilunar Period Is Determined by Counting Circadian Clock Cycles in the Marine Midge Clunio Marinus Supplemental material, sj-jpg-7-jbr-10.1177_07487304241249516 for The Free-Running Circasemilunar Period Is Determined by Counting Circadian Clock Cycles in the Marine Midge Clunio Marinus by Jule Neumann, Dharanish Rajendra and Tobias S. Kaiser in Journal of Biological Rhythms

sj-jpg-8-jbr-10.1177_07487304241249516 – Supplemental material for The Free-Running Circasemilunar Period Is Determined by Counting Circadian Clock Cycles in the Marine Midge Clunio Marinus Supplemental material, sj-jpg-8-jbr-10.1177_07487304241249516 for The Free-Running Circasemilunar Period Is Determined by Counting Circadian Clock Cycles in the Marine Midge Clunio Marinus by Jule Neumann, Dharanish Rajendra and Tobias S. Kaiser in Journal of Biological Rhythms

sj-jpg-9-jbr-10.1177_07487304241249516 – Supplemental material for The Free-Running Circasemilunar Period Is Determined by Counting Circadian Clock Cycles in the Marine Midge Clunio Marinus Supplemental material, sj-jpg-9-jbr-10.1177_07487304241249516 for The Free-Running Circasemilunar Period Is Determined by Counting Circadian Clock Cycles in the Marine Midge Clunio Marinus by Jule Neumann, Dharanish Rajendra and Tobias S. Kaiser in Journal of Biological Rhythms

sj-xlsx-2-jbr-10.1177_07487304241249516 – Supplemental material for The Free-Running Circasemilunar Period Is Determined by Counting Circadian Clock Cycles in the Marine Midge Clunio Marinus Supplemental material, sj-xlsx-2-jbr-10.1177_07487304241249516 for The Free-Running Circasemilunar Period Is Determined by Counting Circadian Clock Cycles in the Marine Midge Clunio Marinus by Jule Neumann, Dharanish Rajendra and Tobias S. Kaiser in Journal of Biological Rhythms

sj-xlsx-3-jbr-10.1177_07487304241249516 – Supplemental material for The Free-Running Circasemilunar Period Is Determined by Counting Circadian Clock Cycles in the Marine Midge Clunio Marinus Supplemental material, sj-xlsx-3-jbr-10.1177_07487304241249516 for The Free-Running Circasemilunar Period Is Determined by Counting Circadian Clock Cycles in the Marine Midge Clunio Marinus by Jule Neumann, Dharanish Rajendra and Tobias S. Kaiser in Journal of Biological Rhythms
