# Supplementary material for: The Free-Running Circasemilunar Period Is Determined by Counting Circadian Clock Cycles in the Marine Midge Clunio Marinus
Source: J Biol Rhythms. 2024 May 19;39(4):379–91. doi: 10.1177/07487304241249516 (PMC11292968; doi:10.1177/07487304241249516)
Supplement: sj-docx-1-jbr-10.1177_07487304241249516 – Supplemental material for The Free-Running Circasemilunar Period Is Determined by Counting Circadian Clock Cycles in the Marine Midge Clunio Marinus [file sj-docx-1-jbr-10.1177_07487304241249516.docx]

## Supplementary Figures

**Figure S1.**Time series data and analysis of the free-running circasemilunar emergence rhythm of *C. marinus* under LD 12:12. Experiments were conducted in 2020 (a,e,i), 2021 (b,f,j) and 2023 (c,g,k and d,h,l). For time series analysis, only the colored bars were considered. (a-d) Daily number of emerged midges after release into circasemilunar free-run and LD 12:12. In one replicate (b) a phase shift was observed (indicated by gray arrow), likely due to vibrations coming from construction works. (e-h) Auto-correlation of time series. The confidence interval was set to 0.95 and is indicated as the gray dashed line. (i-l) Lomb-Scargle periodograms with alpha of 0.05 indicated as gray dashed line. (m) Significant circasemilunar periods calculated with meta2d() are displayed. Colors of dots correspond to the colors of the time series in a-l.

**Figure S2.** Time series data and analysis of the free-running circasemilunar emergence rhythm of *C. marinus* under LD 11:11. Experiments were conducted in 2020 (a,d,g), 2021 (b,e,h) and 2023 (c,f,i). For time series analysis, only the colored bars were considered. (a-d) Daily number of emerged midges after release into circasemilunar free-run and LD 11:11. (d-f) Auto-correlation of time series. The confidence interval was set to 0.95 and is indicated as the gray dashed line. (g-i) Lomb-Scargle periodograms with alpha of 0.05 indicated as gray dashed line. (j) Significant circasemilunar periods calculated with meta2d() are displayed. Colors of dots correspond to the colors of the time series in a-i.

**Figure S3.** Time series data and analysis of the free-running circasemilunar emergence rhythm of *C. marinus* under LD 13:13. Experiments were conducted in 2020 (a,d,g), 2021 (b,e,h) and 2023 (c,f,i). For time series analysis, only the colored bars were considered. (a-d) Daily number of emerged midges after release into circasemilunar free-run and LD 13:13. (d-f) Auto-correlation of time series. The confidence interval was set to 0.95 and is indicated as the gray dashed line. (g-i) Lomb-Scargle periodograms with alpha of 0.05 indicated as gray dashed line. (j) Significant circasemilunar periods calculated with meta2d() are displayed. Colors of dots correspond to the colors of the time series in a-i.

**Figure S4.**Time series data and analysis of the free-running circasemilunar emergence rhythm of *C. marinus* under LD 14:14. The experiment was conducted in 2020 (a-c). For time series analysis, only the colored bars were considered. (a) Daily number of emerged midges after release into circasemilunar free-run and LD 14:14. (b) Auto-correlation of time series. The confidence interval was set to 0.95 and is indicated as the gray dashed line. (c) Lomb-Scargle periodogram with alpha of 0.05 indicated as gray dashed line. (d) There is no significant circasemilunar period calculated with meta2d().

**Figure S5.** Time series data and analysis of the free-running circasemilunar emergence rhythm of *C. marinus* under LD 15:15. The experiment was conducted in 2021 (a-c). For time series analysis, only the colored bars were considered. (a) Daily number of emerged midges after release into circasemilunar free-run and LD 15:15. (b) Auto-correlation of time series. The confidence interval was set to 0.95 and is indicated as the gray dashed line. (c) Lomb-Scargle periodogram with alpha of 0.05 indicated as gray dashed line. (d) There is no significant circasemilunar period calculated with meta2d().

**Figure S6.** Circadian and circasemilunar emergence rhythm under constant light of a *C. marinus* population from Northern Spain. Data from Neumann (1976). (a) The circadian emergence rhythm free-runs under constant light with a period longer than 25 hours. Missing data (NA) is encoded in red. (b) The number of emerged midges per hour for two circasemilunar emergence peaks (peak I: day 1-7 and peak II day 13-19). (c) The circasemilunar emergence rhythm is synchronized under constant light for at least two peaks. Moonlight (dim night light indicated by arrows) was used to entrain the circasemilunar clock. (d) The LS periodogram indicates rhythmicity of circadian emergence for both emergence peaks (peak I: $\tau_{circadian}\text{=23.86h,}$ p=1.14e-08; peak II: $\tau_{circadian}\text{=27.83h,}$ p=1.31e-04). (e) meta2d() detects a significant circadian period for both emergence peaks.
