# Supplementary figures and images for: The Free-Running Circasemilunar Period Is Determined by Counting Circadian Clock Cycles in the Marine Midge Clunio Marinus
Source: J Biol Rhythms. 2024 May 19;39(4):379–91. doi: 10.1177/07487304241249516 (PMC11292968; doi:10.1177/07487304241249516)

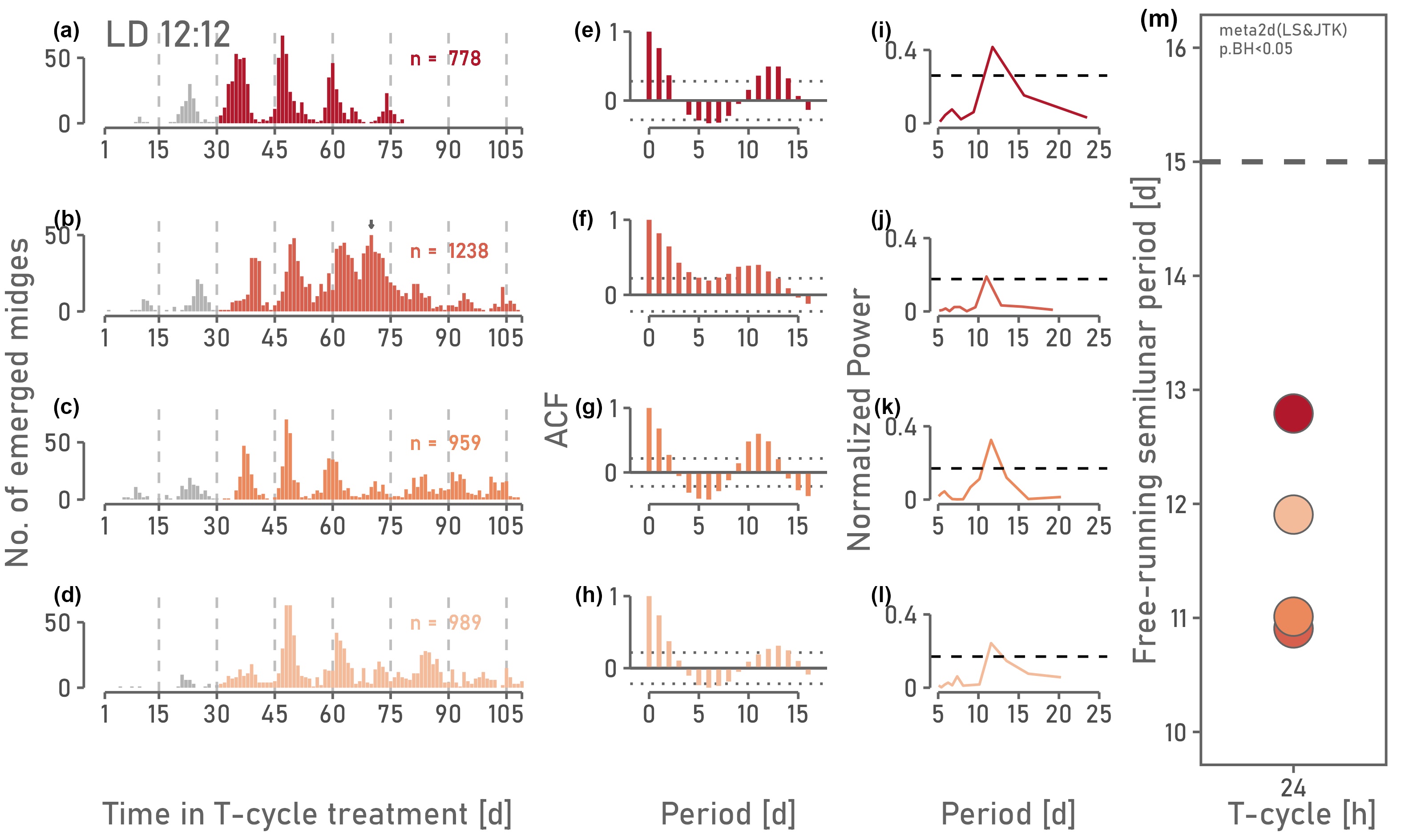

Supplement: sj-jpg-4-jbr-10.1177_07487304241249516 – Supplemental material for The Free-Running Circasemilunar Period Is Determined by Counting Circadian Clock Cycles in the Marine Midge Clunio Marinus [file sj-jpg-4-jbr-10.1177_07487304241249516.jpg]

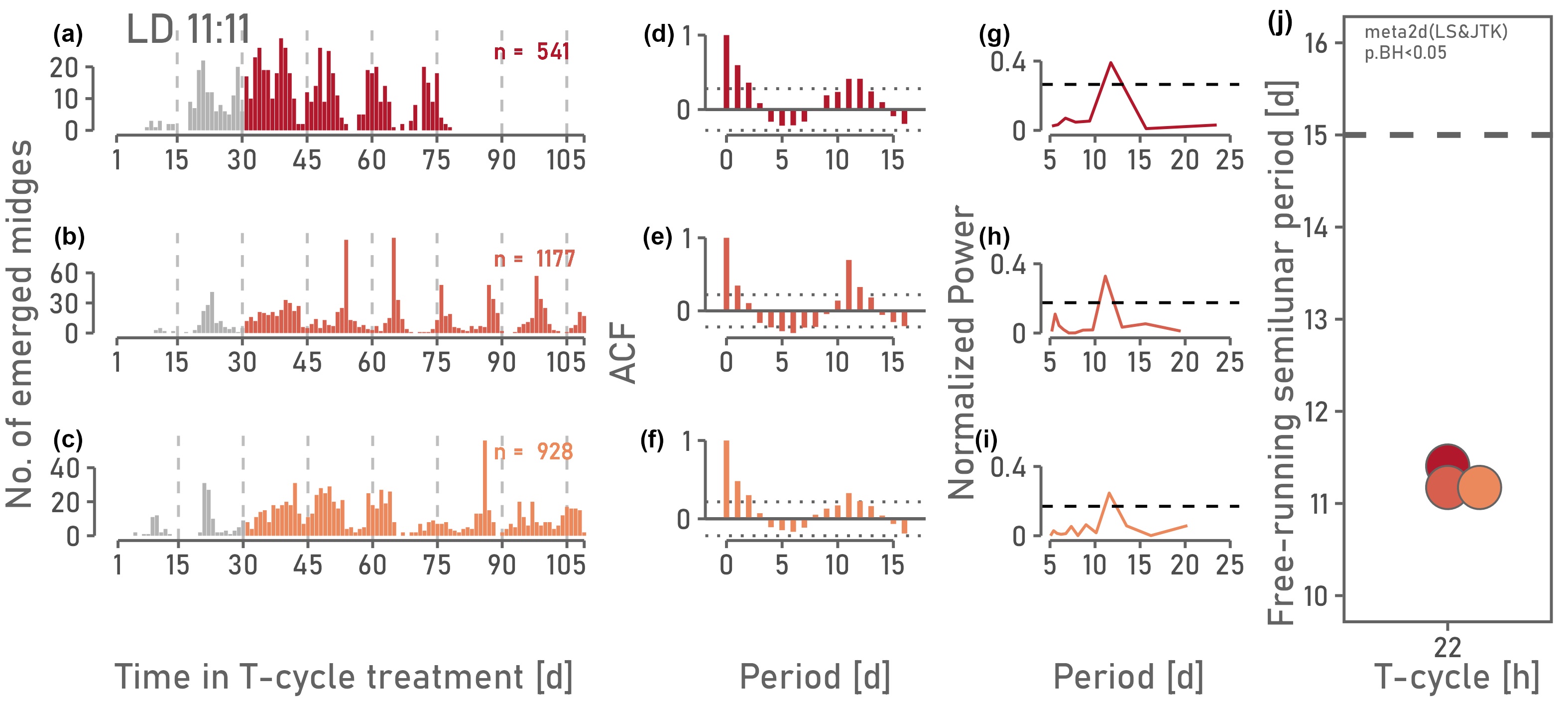

Supplement: sj-jpg-5-jbr-10.1177_07487304241249516 – Supplemental material for The Free-Running Circasemilunar Period Is Determined by Counting Circadian Clock Cycles in the Marine Midge Clunio Marinus [file sj-jpg-5-jbr-10.1177_07487304241249516.jpg]

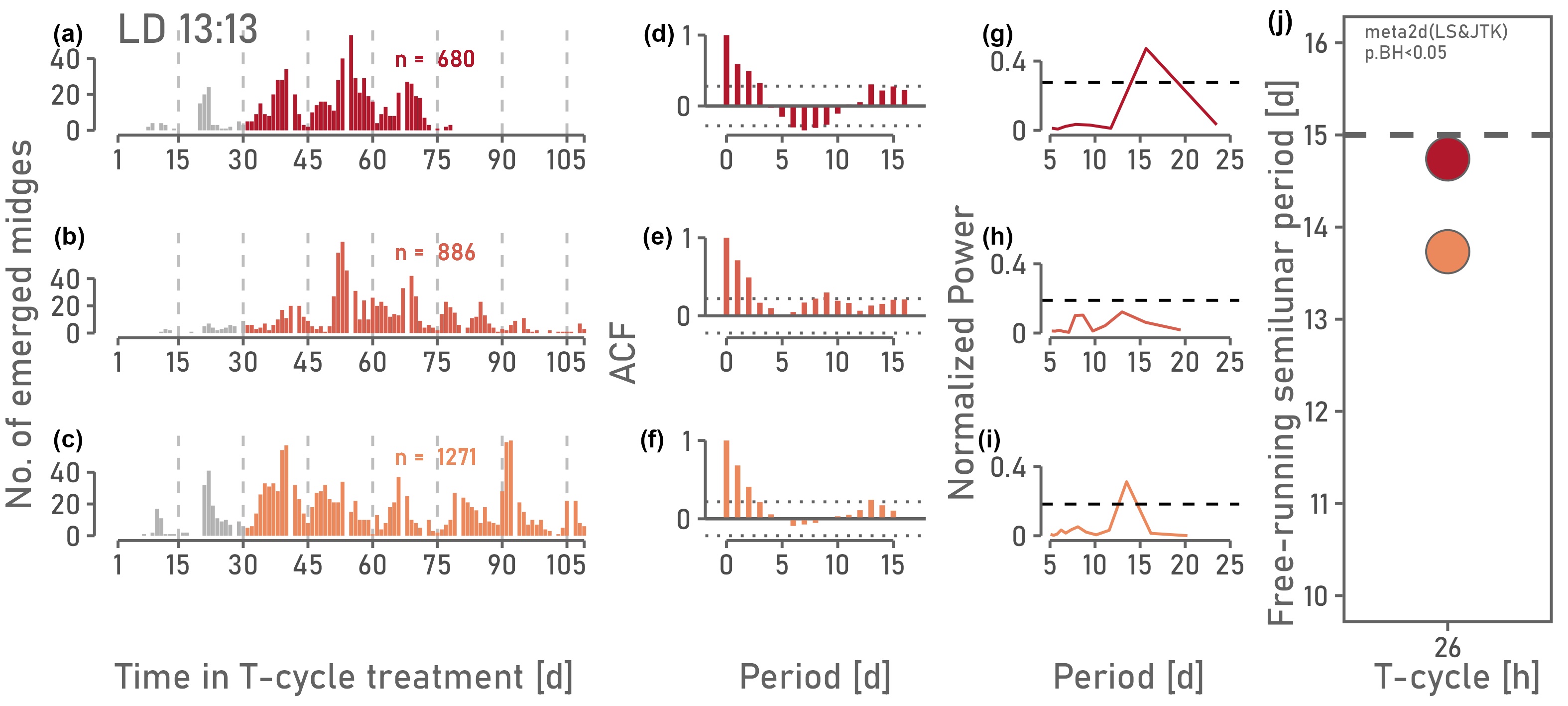

Supplement: sj-jpg-6-jbr-10.1177_07487304241249516 – Supplemental material for The Free-Running Circasemilunar Period Is Determined by Counting Circadian Clock Cycles in the Marine Midge Clunio Marinus [file sj-jpg-6-jbr-10.1177_07487304241249516.jpg]

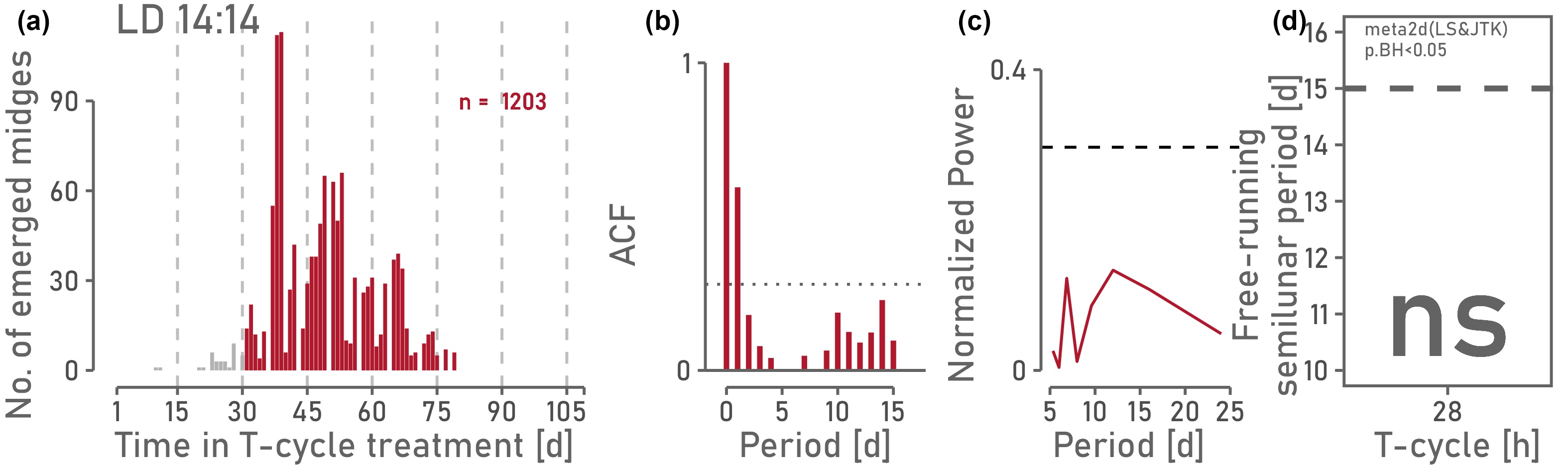

Supplement: sj-jpg-7-jbr-10.1177_07487304241249516 – Supplemental material for The Free-Running Circasemilunar Period Is Determined by Counting Circadian Clock Cycles in the Marine Midge Clunio Marinus [file sj-jpg-7-jbr-10.1177_07487304241249516.jpg]

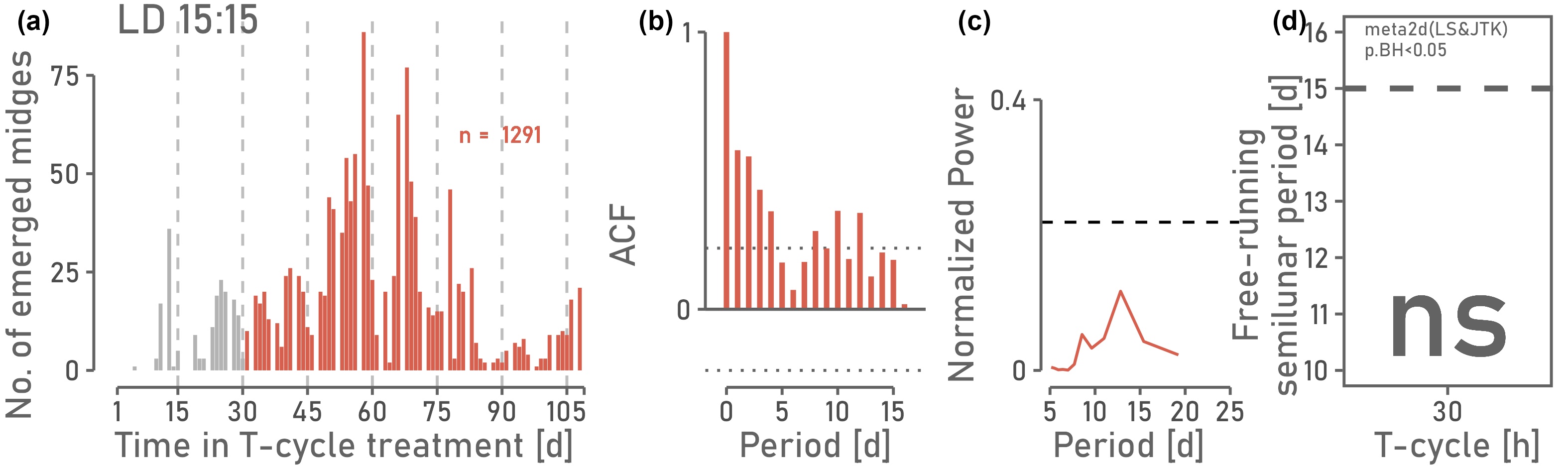

Supplement: sj-jpg-8-jbr-10.1177_07487304241249516 – Supplemental material for The Free-Running Circasemilunar Period Is Determined by Counting Circadian Clock Cycles in the Marine Midge Clunio Marinus [file sj-jpg-8-jbr-10.1177_07487304241249516.jpg]

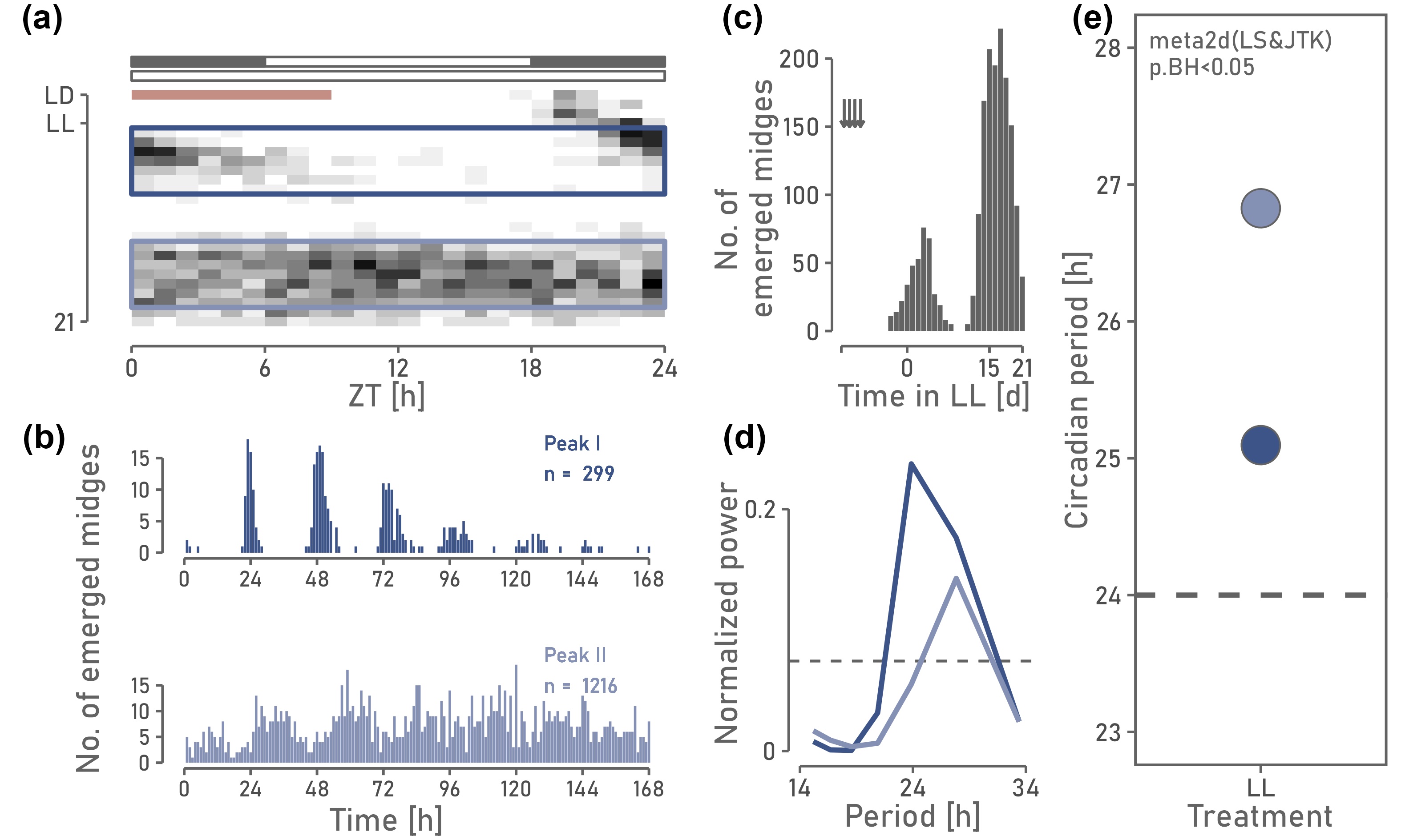

Supplement: sj-jpg-9-jbr-10.1177_07487304241249516 – Supplemental material for The Free-Running Circasemilunar Period Is Determined by Counting Circadian Clock Cycles in the Marine Midge Clunio Marinus [file sj-jpg-9-jbr-10.1177_07487304241249516.jpg]
